# Practical Application of Circulating Tumor-Related DNA of Human Papillomavirus in Liquid Biopsy to Evaluate the Molecular Response in Patients with Oropharyngeal Cancer

**DOI:** 10.3390/cancers15041047

**Published:** 2023-02-07

**Authors:** Agnieszka M. Mazurek, Tomasz W. Rutkowski

**Affiliations:** 1 Center for Translational Research and Molecular Biology of Cancer, Maria Sklodowska-Curie National Research Institute of Oncology Gliwice Branch, Wybrzeże Armii Krajowej 15, 44-102 Gliwice, Poland; 2 I Radiation and Clinical Oncology Department, Maria Sklodowska-Curie National Research Institute of Oncology Gliwice Branch, Wybrzeże Armii Krajowej 15, 44-102 Gliwice, Poland; 3 Radiotherapy Department, Maria Sklodowska-Curie National Research Institute of Oncology Gliwice Branch, Wybrzeże Armii Krajowej 15, 44-102 Gliwice, Poland

**Keywords:** radiotherapy, chemotherapy, oropharyngeal cancer, human papillomavirus, circulating tumor HPV DNA, liquid biopsy, molecular response

## Abstract

**Simple Summary:**

Oropharyngeal cancer (OPC) is one of the most common cancers in the head and neck region. The incidence of OPC is still growing, mostly due to the human papillomavirus (HPV), which has become the dominant etiological factor. HPV16 is the most relevant genotype for OPC, whereas other high-risk genotypes are rare and may be associated with a worse prognosis. The aim of this article is to discuss the molecular response (MR) obtained by assessing circulating tumor-related HPV DNA (ctHPV) in a liquid biopsy (LB). This quick and simple diagnostic tool reflects the presence of the tumor and thus, can support some clinical decisions. We provide a comprehensive overview of the clinical benefits of the blood-based molecular response, focusing on the decision-making process of the tumor response during the therapy, assessment of early treatment results, and post-treatment follow-up in patients with HPV-related OPC.

**Abstract:**

Recent findings have shown that human papillomavirus (HPV) DNA is present in the blood as a tumor-specific biomarker (circulating tumor-related HPV; ctHPV) in patients with HPV-related oropharyngeal cancer (HPV-related OPC). The molecular response (MR) in patients with HPV-related OPC can be defined as the change in the number of ctHPV copies in relation to its initial quantity. The optimal model for assessing the MR using a liquid biopsy (LB) should be based on the E6/E7 sequences of the viral genome. MR assessment can help to evaluate the intensity of ongoing treatments in relation to the tumor response. The evaluation of the residual disease at the end of therapy may also be performed by MR assessment. If a partial MR (pMR) is found, caution is indicated and a subsequent LB should be considered, due to the likelihood of disease progression. Complete radiological and clinical responses together with a complete MR (cMR) convincingly indicate a low risk of treatment failure. Moreover, molecular recurrence (Mrec) during a follow-up, confirmed in two consecutive assays, even despite the lack of any other clinical or radiological symptoms of progression, indicates patients at high risk of disease recurrence. In conclusion, MR by ctHPV assessment may hasten the early detection of disease progression, at any stage of the management of the patient with HPV-related OPC.

## 1. Introduction

Recognizable and well-known cancer biomarkers, although desirable, are scarce in the oncological diagnostics. Eligible biomarkers not only play important role in the detection of neoplasmatic diseases, but also help to monitor both the active disease (through the therapeutic process) and cured patients (during surveillance). The cancer cell differs from its normal archetype in many aspects and, at different levels of magnification, morphological, cytogenetic, and genetic changes can be observed. The genetic changes found in the blood come exclusively from the tumor cell [1,2,3,4] and constitute the circulating tumor DNA (ctDNA) fraction [5,6]. It should be noted that ctDNA is a component of the so-called total circulating cell-free DNA (cfDNA). DNA from normal cells represents the main fraction of total cfDNA and is a common finding in the healthy population [7]. This innovative molecular analysis of ctDNA, based on blood and generally referred to as a liquid biopsy (LB), strongly supports the existing classical diagnostics of solid tumors [8,9,10]. The proposed candidate for ctDNA is a fragment of the HPV genome—denoted in this review by circulating tumor-related HPV (ctHPV)—as a cause–effect agent and specific only to patients with HPV-related cancer [11].

The percentage of HPV-related oropharyngeal cancer (HPV-related OPC) has increased significantly over the past twenty years, with sharper and faster rates of increase reported in Western Europe compared to North America [12]. An increase in over 16% was observed between 2000 and 2006, as well as a 46% increase during 2013–2018 in North-East Italy [13]. The number of patients with HPV-related OPCs between 2000 and 2017 has presented a twofold increase (in Giessen Germany) [14] to threefold increase (in Eastern Denmark) [15].

In this review, we propose a quick and simple diagnostic method for HPV-related OPC, based on a LB and the most-documented HPV16 genotype. We provide a comprehensive overview of the clinical benefits of blood-based ctHPV analysis, focusing on the decision-making process of the tumor response during therapy, assessment of early treatment results, and post-treatment follow-up.

## 2. Clinical Aspects of HPV Genotypes and Prognostic Role of HPV16 

The human papillomaviruses are a large and diverse group of viruses with five main genera: *Alphapapillomavirus*, *Betapapillomavirus*, *Gammapapillomavirus*, *Mupapillomavirus*, and *Nupapillomavirus*. It has been estimated that there are at least 400 different types of HPV, and the number of newly discovered types continues to increase thanks to rapidly advancing metagenomic sequencing technologies [16,17]. From a clinical point of view, the distinction of high-risk (HR) and low-risk (LR) strains is significant, as HR strains present oncogenic potential and are responsible for cancer in humans [18]. Among the HR genotypes, HPV16 is the most common virus, accounting for almost 90% of all HPV-related OPC (see Table 1). The remaining HR types account for around 10–13% of HPV-related OPCs, where HPV35 and HPV33 occur at higher frequencies (4.1% and 3.7%, respectively), compared to HPV18 (3%). Other HR types are very rarely detected (e.g., 0.9% [19]). The presented contribution of individual genotypes to the oncogenic development of OPC indicates that, apart from the common HPV16 genotype, the HPV33 and HPV35 genotypes are mainly involved in the development of HPV-related OPC.

With advances in HPV genotyping, more information has been gained from studies comparing the clinical significance of HPV16 with other HR genotypes. In most reported series, clinical factors were similarly distributed between HPV16 and other HR HPVs, including p16 expression, smoking, alcohol use, race, age, and sub-site of the tumor [26,32]. Varier et al. have described a slightly lower age and lower clinical T-stage for HPV16-related OPC compared to other HR HPV-related OPCs [31]. Moreover, Shenker et al. has found that patients with HPV16-related OPC were younger than those with other HR HPVs (mean age: 58.9 vs. 63.7 years) [25]; however, this difference was not confirmed by Ziai et al. [30].

The presence of HPV in tumors was first considered a strong positive prognostic factor for patients suffering from squamous cell carcinoma of the head and neck (HNSCC) [33,34]. Since then, the dynamic development of genetics and genotyping studies has brought additional information to this field. No et al. have indicated that HPV18 positivity, together with old age and advanced T-stage, were independent prognostic factors for poor outcomes in patients with tonsillar carcinoma [35]. An analysis based on The Cancer Genome Atlas (TCGA Research Network) has shown that the 3-year overall survival (OS) was significantly worse for the cohort with other HR HPVs (49%) than those with HPV16 (88%) [32]. Yoo et al. pointed out that, in a group of 152 patients with locally advanced HNSCC, those with HPV18 presented significantly poorer OS and disease-free survival (DFS) [36]. Mazul et al. reported 5-year OS rates of 71.4% and 57.1% (*p* = 0.01) for patients with HPV16-related OPC and those with other HR HPVs, respectively. Notably, the 5-year OS for HPV-unrelated OPC was 50%, which was similar to the OPC with other HR HPVs [26]. In contrast to the above, one study have presented a trend of a worse 5-year OS and PFS for HPV16-related cases compared to OPC cases with other HR HPVs [30]. In a large study by Garset-Zamani et al., no difference in survival was observed between HPV16 and other HR HPVs; however, when sub-grouping the other HR HPV groups into HPV33 (n = 57), HPV35 (n = 26), and “other genotypes” (n = 24), a significantly worse OS in the “other genotypes” group was presented [28]. In contrast, two studies excluded any difference in the genotype’s effect on survival [25,31].

The true mechanisms underlying the poorer results for OPC patients with HR HPVs other than HPV16 have not yet been fully elucidated, although a possible role of the immune system has been highlighted. Dramatically inferior outcomes for patients with HPV33-related HNSCC compared to HPV16-related HNSCC have been noted by Chatfield-Reed et al. [37], who paid attention to the specific signatures of APOBEC enzymes (a family of cytidine deaminases that promote the conversion of deoxycytidine to deoxythymidine), which have previously been shown to regulate innate antiviral immunity, including that for HPV [38]. The enrichment of the APOBEC signature in HPV16-related tumors may reflect a past infection or may affect the post-transcriptional regulation important for the immune reaction during the therapeutic response. It has also been shown that CD8+ cytotoxic T-cell infiltration, which is a prognostic factor for a better response [39], was reduced in HPV33-related compared to HPV16-related tumors [37]. Thus, the distinct genomic and immune landscapes associated with the HPV16 and HPV33 genotypes may contribute to their different clinical outcomes.

In summary, HPV16 is the most relevant HR genotype for OPC, whereas other HR genotypes, such as HPV33, HPV35, and HPV18, are rare and may be associated with a worse prognosis. The survival benefit of an HPV-related patient may be genotype-specific, so care should be taken in extending the knowledge based on HPV16 to other HR genotypes in patients with HPV-related OPC.

## 3. Clinical Factors Affecting the Quantity of ctHPV16 

For patients with HPV16-related OPC, the viral load (VL) of ctHPV16 is assessed as the number of viral particles per milliliter of fluid [40,41]. The HPV VL is the number of HPV DNA copies in a tissue sample (tumor VL, tVL; in unit copies/genome) or in the bloodstream (blood VL, bVL; in unit copies/mL). Several factors may significantly influence the HPV VL. For patients with HPV-related OPC, the bVL is strongly positively correlated with the tVL. A higher tVL increases the probability of detecting ctHPV16 in the blood [42]. The total tumor volume (TV), composed of the primary TV and the volume of involved regional nodes, also influences the bVL. The larger the TV, the greater the number of HPV copies in the bloodstream [41,43,44,45]. Despite this, no clear correlation between the bVL and the T- or N-stage has been found. Some authors have reported that the pretreatment bVL was directly proportional to the N-stage, while no such trend was observed for the T-stage [40,41,46]. Chera et al. have observed a higher level of bVL before treatment in patients with T2 tumors, compared to T0/T1 tumors. Surprisingly, patients with T3/T4 tumors had significantly lower levels of the bVL, compared to patients with T2 tumors [46]. A possible explanation for this observation is that the TNM system for OPC does not reflect the volume of the tumor precisely, instead describing the linear dimension of the tumor. These differences between the dimension and volume increase in a cubic manner, and they are more noticeable for larger tumors. Nevertheless, many studies have described a relationship between the overall stage of the disease and the bVL, which may indicate that this parameter more accurately corresponds to the total TV than simply T or N. There also exists a proportional relationship between the bVL and distant metastasis (DM), confirming that the magnitude of the disease corresponds to the number of viral copies in the blood [43].

Some differences between males and females regarding the VL have also been observed in HPV-related OPCs. Men with detectable ctHPV16 mainly showed N2/N3 diseases and high tVLs, while those with no ctHPV16 showed predominantly N0/N1 diseases and low tVLs. For women, multiple regression revealed that both an advanced T-stage and high tVLs significantly increased the detection of ctHPV16 [42]. A particularly high tVL was found in non-smoking women. Interestingly, an in vitro model indicated that the glucocorticoid hormone significantly stimulated viral DNA replication and may increase the level of viral DNA replication by at least 33% [47].

In summary, the VL describes the amount of viral DNA in tissue or in the blood. In general, the larger the tumor mass (T-stage and N-stage), the higher the number of copies of the viral DNA in the tumor and, thus, the larger the blood-based VL.

## 4. Proposal for an LB-Based HPV Diagnostics Regimen

### 4.1. E6/E7 Sequence as the ctDNA Marker

It has been successfully proven that ctHPV is correlated with the existence of cancer and is not detectable in healthy people or in patients with an HPV-unrelated tumor [45,48]. The development of HPV-dependent cancer is a multi-stage process including E6/E7 expression, the integration of HPV DNA fragments into the host cell DNA, and the progression of numerical and structural changes in chromosomes [49,50], finally producing aggressive tumor characteristics [51,52]; however, in HPV-related OPCs, evidence of viral integration has been detected in 74% of patients, with the remaining tumors harboring the episomal form [53]. Of the three viral oncoproteins—namely, E5, E6, and E7—which are necessary for neoplastic transformation [54,55,56,57], E6 and E7 have been found to be retained and expressed as virus-host fusion transcripts in all virus-positive samples [58]. As these viral sequences are retained by the tumor cell until the cancer is advanced, they may be a marker of cancerous cells at almost every stage of the disease. The optimal model in the liquid biopsy is the use of E6 and/or E7 oncogenes for the detection and monitoring of neoplastic diseases [59]. As the tumor progresses, cells undergo apoptosis and necrosis, and its DNA enters the bloodstream, along with the E6 and E7 DNA sequences. The fragments E6 and E7 of HPV DNA (ctHPV) may serve as tumor identifiers (ctDNA). The detection of E6/E7 in the blood of patients with HPV-dependent OPC has become the basis of the concept whereby HPV DNA in the bloodstream (ctHPV) represents a tumor-derived biomarker [60,61,62].

### 4.2. ddPCR or qPCR in the First Line of OPC Diagnostics

Suitable methods for HPV detection have become increasingly complex. Although detection by the polymerase chain reaction (PCR), quantitative real-time PCR (qPCR), reverse transcription PCR (RT-PCR), immunohistochemistry (IHC), in situ hybridization (ISH), or a combination of these methods are appropriate when considering tissue samples [63,64], next-generation sequencing (NGS) and digital droplet PCR (ddPCR) are appropriate for LB-based detection [65]. To reveal the presence of a unique cancer-related sequence to detect oncogenic viruses, qPCR or ddPCR have been shown to be optimal, due to their relatively low cost and the sufficiency of resources in most laboratories [45,66].

All three methods—namely, qPCR, ddPCR, and NGS—are based on PCR technology. A common feature of qPCR and ddPCR is the use of fluorescent probes and primers for the detection of a specific fragment, where the developed set (probe and primers) can be used interchangeably. The difference is that ddPCR is stoichiometrically designed, such that there is only one template per droplet. As such, both the wild and mutant form initially have the same chance of amplification; in other words, there is no possibility of faster amplification of one of them, which would result in the inhibition of the reaction by the amplified product. This is important in mutation testing, where it is assumed that there is one mutant and one normal allele in the droplet. However, when testing viral DNA—the number of copies of which often exceeds the number of normal alleles (one of which serves as a control) by tens to several hundreds of times—stoichiometric matching is not possible. Thus, the stoichiometric ratio of virus copies to control alleles may be under- or over-estimated in droplet PCR. For qPCR, the stoichiometric ratio is not important, but it is undesirable to amplify the control gene too quickly. This can lead to the accumulation of the control product, an excess of which can inhibit the amplification of the target gene in situations where there is not enough of it; hence, qPCR is a less-sensitive technique [67]. NGS technology, similarly to ddPCR, is based on the amplification in droplets, but detection is based on sequencing of the chosen targets (or whole sequencing). Using the NGS technology, it is possible to identify all high-risk types in one sample, and additionally allows for the examination of the degree of integration of viral DNA with human DNA [64].

The diagnostic accuracy regarding ctHPV, almost exclusively based on ddPCR or qPCR and with the detection of E6/E7 fragments, has been discussed in a few meta-analyses. At the first diagnosis, ctHPV detection in pre-treatment blood samples showed an overall sensitivity of 81% [68,69], and an overall specificity of 95% [69] to 98% [68]. The pooled positive likelihood ratio (PLR) was found to be 23.24, while the negative likelihood ratio (NLR) was 0.17 [68]. It is worth noting that the high heterogeneity at the first diagnosis for sensitivity (I2 = 88%), but not specificity (I2 = 0%) was established [68]. Finally, Wuerdemann et al. have documented that such discrepancies are the result of the inferior sensitivity of the qPCR method (65%), compared to the ddPCR method (92%); whereas the specificity was very high for both methods (100% for qPCR, 95% for ddPCR). The AUC value for ddPCR was higher than that for qPCR (0.97 and 0.89, respectively) at the pre-treatment diagnosis [68]; however, ddPCR was less specific for recurrence detection. Campo et al. have provided comprehensive data on the use of ctHPV for the post-treatment surveillance of HPV-related patients [70]. In their meta-analysis, the diagnostic pooled accuracy of ctHPV achieved a DOR score of 371.66 (high values suggest adequate diagnostic accuracy), while the individual study results indicated that the two ddPCR-based studies had a low DOR, and the two qPCR-based studies had a high DOR. Nevertheless, the pooled PLR of 62.53 and NLR ratio of 0.05 demonstrated the adequate diagnostic utility of ctHPV for follow-ups [70].

**Figure 1:**
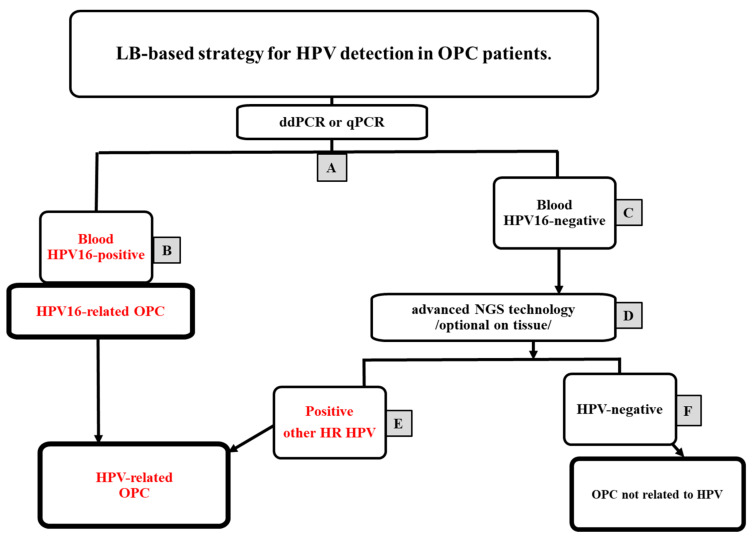
The identification of HPV-related cancer by liquid biopsy in the primary diagnosis of a patient with oropharyngeal cancer (OPC). (**A**) In the first line of molecular diagnostics, the use of a fast, simple, and inexpensive ddPCR or qPCR technique enables the detection of the most common HPV16 virus in the LB; (**B**) a positive HPV16 result indicates an HPV16-related OPC; (**C**) a negative ddPCR/qPCR result is an indication for in-depth molecular diagnostics, due to the possibility of other HR HPVs; (**D**) the use of more expensive and complex NGS-based molecular diagnostics gives the best chance of detecting other types, preferentially performed in a LB (or optionally on scraps); (**E**) a positive HR ctHPV result in NGS indicates an HPV-related OPC; and (**F**) a negative NGS test result indicates a disease independent of the HPV etiology.

As HPV16 is known to be the most prevalent HPV genotype in OPC, it is better to focus on the detection of the HPV16 genotype by LB, based on qPCR/ddPCR, in the first line of diagnostics (Figure 1).

Since there are currently no commercial tests using LB for OPC patients, the laboratory can use a molecular test developed in-house to make decisions regarding patient care [64,71]. The implementation and use of such a test can be considered acceptable after full rigorous validation, including a demonstration of clinical usefulness [72]. In the context of the ctHPV16 test, the pre-treatment copy number determination is of prognostic importance, as patients with adverse clinical risk factors—such as smoking history of more than ten tobacco pack years (TPY) or T4—tend to have lower baseline levels of ctHPV16 than patients with a low clinical risk (≤10 TPY and <T4). As demonstrated by Chera et al., patients with >200 copies/mL of ctHPV16 in the pre-treatment and rapid clearance during treatment had 100% regional disease-free survival at 2 years, compared to those with an unfavorable profile of ≤200 copies/mL; these results strongly promote the use of such an approach [46]. The application of the test must be accompanied by high-level knowledge and expertise regarding the details of ddPCR and qPCR technological advances, including their application to viral identification [67].

### 4.3. NGS in the Second Line of OPC Diagnostics

The discrepancies between ddPCR and qPCR in the sensitivity of detection of ctHPV16 can be adjusted by using NGS in the second line of LB-based diagnostics, allowing for the further detection of other genotypes. While the importance of these genotypes in the survival is not yet clear, their use as biomarkers for recurrence has been well-documented [58,73,74]. NGS can also be performed on tissues or biopsies, if available. The use of NGS methods can significantly affect the accuracy of ctHPV diagnostics, both by detecting other types, as well as by identifying the episomal vs. integrative status. The latter aspect is important as the episomal status—by preserving highly immunogenic sequences—may affect the treatment, according to the relative ability to induce the immune system. However, at present, the literature requires more documentation in this line; furthermore, due to the cost and complexity of NGS methodologies, it is worth considering this tool in the context of complementary diagnostics [75,76].

In summary, the E6 and E7 fragments can only be found in a LB in the presence of cancer and, consequently, can be used as tumor markers. In the first line of diagnostics, the detection of E6/E7 fragments of the most common type (HPV16) can be efficiently performed using the ddPCR or qPCR methods; NGS, as the next step, is expected to complement such a diagnosis.

## 5. ctHPV16-Based Molecular Response in LB

Determination of ctHPV16 in the LB, in order to monitor the effectiveness of treatment in patients with HPV-related OPC, seems to be a particularly useful diagnostic tool. A LB is relatively easy and safe, and it may be subsequently repeated. Serial measurements of the amount of ctDNA show its change, which can be used to determine the molecular response (MR) to ongoing therapy, or to confirm the residual tumor after completion of the treatment [77].

The MR of patients with HPV16-related OPC can be defined by the change in number of ctHPV16 copies in relation to the initial quantity [78]. If the number of copies drops to zero, complete MR (cMR) has been obtained. Decreasing but still detectable ctHPV represents a partial molecular response (pMR). Molecular recurrence (Mrec) is the re-appearance of ctHPV16 in a patient who previously showed a cMR after effective therapy.

### 5.1. ctHPV16 Assay Prior to Treatment—Complementation of the Primary Diagnosis

Patients with HPV-related OPC tend to respond better to treatment and present a higher overall survival (OS) rate [5,79,80]. Consequently, for HPV-related OPC, a distinct TNM classification has been adopted, which requires the assessment of OPC etiology prior to any treatment decision. The primary diagnosis of OPC includes determining the tumor HPV status by immunohistochemistry (IHC) against the protein p16 and/or HPV PCR tissue testing. When these two methods were combined, the sensitivity and specificity of HPV etiology increased to 93% and 86%, respectively [69,81]. The protein p16 is often over-expressed in HPV-related OPC and so, it has been widely used as a surrogate marker for HPV status; consequently, p16-positive OPC is typically considered as HPV-related. Despite this, p16 is not always highly reliable or specific [82,83,84], and its over-expression does not necessarily correspond to an HPV-positive status. Wagner et al. have observed contradictory results in approximately 11% of all samples analyzed for both PCR and IHC [21]. Hashida et al. have reported that 28% of the p16-positive cases were HPV DNA-negative, according to PCR [85]. Mazul et al. have shown that approximately 12% of OPC cases negative for p16 expression were positive for HPV16, as assessed by PCR [26]. Overall, the specificity of p16 does not exceed 83% due to a different, HPV-independent mechanism for the induction of p16 over-expression during tumorigenesis [81,86]. The suboptimal usefulness of p16 alone as a surrogate marker for HPV etiology has also been noted in studies on survival or the response to therapy [21,85,87]. The prognosis for OPC with p16-positive and HPV DNA-negative tumors was significantly worse than that for patients with p16-positive and HPV DNA-positive tumors [21]. Similarly, Hashid et al. have shown that HPV DNA-positive patients with high VLs had improved OS and PFS, whereas patients with either low VLs or no viral genome had a poor prognosis, even when p16 immunostaining yielded positive results [85]. In addition, the p16 protein cannot be used to distinguish the most common virus type (i.e., HPV16) from other HR HPV genotypes, what may be important for risk stratification. 

It seems reasonable to support molecular pathological diagnosis by conducting a LB in the situations where the amount of the tissue sample is insufficient to confirm the HPV etiology, or when biopsies would require repetitions, due to the localization or composition of the tumor (Table 2A).

### 5.2. ctHPV16 Assay during Treatment—Prediction of Good Response

As there is a considerably more favorable prognosis for HPV-related OPC than HPV-unrelated cases, the possibility of de-escalating therapy to reduce treatment-related toxicity has been considered. While many prospective trials considering this aspect are ongoing, due to the lack of convincing results, the treatment strategy for HPV-related OPC has not yet changed [88]. The selection of potential treatment responders and non-responders is crucial not only for the stratification for de-escalation trials, but also for an immediate response in the event of a lack of remission or progressive disease during the ongoing treatment.

The standard treatment approach for patients suffering from HPV-related OPC consists of either seven weeks of RT (70 Gy) combined with concurrent cisplatin (radiochemotherapy, CHRT), or surgery followed by six weeks of adjuvant RT (60 to 66 Gy) with or without cisplatin, depending on various risk factors [89]. To increase the intensity of the treatment, cisplatin-based induction CHT (iCH) can be used. Although iCH has not shown significant superiority to CHRT [90,91], a trend in favor of iCH in high-risk tumors (N2c and N3) has been observed [90,91,92]. Clinical observations have indicated that HPV-related OPC usually presents high nodal involvement and, in selected cases with bulky disease, iCH may be considered [80,93]. Marur et al. have suggested that response of HPV-related tumor to iCH could be used to select patients for a de-escalated radiation dose [94]. However, for more intense CHT, despite the generally good response of HPV-related OPC to cisplatin for the T4 or N3 subsets of patients from this group, a trend towards inferior survival with cisplatin ≤ 200 mg/m^2^ was observed [95]. It has been speculated that TV reduction following iCH predicting the complete pathologic response of the primary tumor may be used as an indicator for less intense subsequent RT. This may imply that subsequent RT and/or surgery might be unnecessary and a follow-up, instead, may be sufficient [96,97]. In such cases, cMR along with radiological remission (RR) and clinical remission may support the decision to stop iCH earlier than after the standard three cycles, and/or to continue the treatment with RT alone instead of CHRT, or hypothetically, to not continue treatment at all. Inukai et al. have reported a case of a 46 year-old man with p16-positive OPC who underwent iCH with clinically significant tumor shrinkage who underwent subsequent surgery. The post-chemotherapy surgical specimen showed pathological complete response with no p16-positive cells. According to the authors, this suggests the possibility of de-escalation in selected cases [97]. Chera et al. have found that the rate of ctHPV16 clearance was correlated with CHRT sensitivity. They used Week four of CHRT as a benchmark timepoint and defined a favorable ctHPV16 clearance profile as an elevated baseline level of ctHPV16 (>200 copies/mL), which was rapidly cleared (>95% clearance of pre-treatment levels by Week four). Patients with such a favorable ctHPV16 clearance profile had 100% regional DFS at the 18th month. Among patients with an unfavorable ctHPV16 clearance profile, those with adverse clinical risk factors (>10 TPY or T4) experienced 35% of the residual or recurrent regional nodal disease at the 18th month, resembling the disease control rates after CHRT for HPV-unrelated OPC [46]. The MR of ctHPV is also of potential importance in assessing the risk of the residual disease in patients undergoing curative surgery. It has been shown that, in patients without pathologic risk factors for recurrence who were observed after surgery, ctHPV rapidly decreased (<1 copy/mL), whereas ctHPV was markedly elevated on post-operative day 1 (>350 copies/mL) in patients with risk factors for macroscopic residual disease, and remained elevated until the adjuvant treatment [98].

It should be stressed that the efficacy of de-escalation has not yet been confirmed in clinical trials for patients with HPV-related OPC. Nevertheless, due to a distinctive sub-group with a worse prognosis (i.e., heavy smokers with T4N3 disease), a well-defined risk sub-group in the HPV-related population poses a challenge for continued de-escalation in clinical trials [99].

In summary (Table 2B), molecular response by ctHPV16 assessment may help to actively monitor the intensity of the ongoing treatment. A complete MR together with a good radiological and clinical response, may in the future support de-escalation approache in selected cases of HPV-related OPC (for instance, in situation of excessive toxicity).

### 5.3. ctHPV16 Assay after Treatment Completion—Early Detection of Treatment Failure 

For patients with HNSCC, the complete response rates after CHRT at the primary site and regional nodes are in the ranges of 35–55% and 40–70%, respectively [100,101]. Surgery is a salvage option for not-cured patients, and it can be assumed that the sooner the treatment failure is detected, the better the results of salvage [102,103]. Salvage surgery, when justified, is of special significance for patients with HPV-related OPC, who are otherwise often healthy and young survivors [103,104]. It should be noted that approximately 60% of the post-operative specimens after salvage surgery did not contain cancer [100,105,106], and the risk of complications was significant [107]. Identifying an appropriate point in time for reliably evaluating the early results of completed treatment is challenging. It has been established that the radiological assessment may be misleading when conducted earlier than the 12th week after the treatment completion, due to treatment-related changes which may mimic a residual tumor. Contrast-enhanced computed tomography (CT) and magnetic resonance imaging (MRI) are frequently used to assess early treatment results. These imaging modalities have distinct limitations, including the accurate identification of the viable tumor within residual masses [108]. PET-CT appears to be the most accurate imaging diagnostic tool for early assessment of the therapy response; however, this also presents certain limitations, due to treatment-related inflammation or infection [101].

It is of special concern that, for patients with HPV-related OPC, the regression of residual lymph nodes may present a prolonged course, and a radiologically evident post-treatment residual mass may not necessarily indicate the presence of active cancer [109]. The residual nodal neck mass in this group of patients may be observed even beyond one year after completion of the treatment [110]. It has been suggested that residual nodal dissection can be avoided for selected patients (N2, HPV-related) with significant lymph node involvement, if they undergo continued imaging surveillance [109]. ctHPV16 assessment may support this decision regarding the optimal time for salvage surgery in such patients. Our previous data showed that, in a group of 24 patients with HPV-related OPC with a partial radiological response (pRR), five patients (28%) had pMR in the LB at 12 weeks after treatment. Cancer cells were found in biopted material from the residual disease in all of them. In contrast, all others with pRR but with cMR showed no cancer cells in the tissue material from the residual mass, and all had a complete response in subsequent imaging examinations. This observation suggests that ctHPV16 assessment along with imaging may be more precise than imaging alone in indicating patients with the residual disease after the completion of the treatment. Some of our patients with residual nodal mass despite cMR underwent nodal disease or biopsies, and no cancer tissue was confirmed in any of them [78]. Moreover, Lee et al. have shown that those who underwent nodal dissection due to 18F-FDG uptake in cervical nodes but with cMR failed to show any histopathologically proven residual tumor tissue [111]. Tanaka et al. have prospectively correlated ctHPV16 and the metabolic response with the treatment failure. After a median follow-up of 21 months, the ctHPV16 and PET-CT had similar negative prognostic values (90% vs. 84%), whereas the positive prognostic value was much higher for ctHPV16 than for PET-CT (100% vs. 50%). Notably, similarly to our findings, six patients who had post-treatment pMR all experienced treatment failure [112]. The differences in tumor-infiltrating lymphocytes between the HPV-related and HPV-unrelated OPC may cause the prolongation of inflammation, which may translate to the especially low (0–30%) positive prognostic value of PET-CT in HPV-related OPC [113,114,115]. Such a relation may suggest the need to increase the positive prognostic value when postponing PET-CT for longer than 12 weeks after completion of the treatment. Liu et al. have compared the positive prognostic value of PET-CT at 12 and 16 weeks after treatment in HPV-related OPC. The positive prognostic value increased from 12% to 33% but remained poor [116]. Tanaka has reported two patients who had cRR in PET-CT but still presented pMR, both of whom showed recurrence during a follow-up. In contrast, none of the patients who had pRR and cMR experienced treatment failure [112].

Due to these observations in addition to radiological findings in clinical practice, for patients with cMR, postponing surgery could be reasonable, while it seems necessary in those with pMR. Tanaka has suggested three categories of treatment failure risk for patients with HPV-related OPC after completion of the treatment: patients with pRR in PET-CT and pMR, discordant results between PET-CT and MR, or cRR in PET-CT together with cMR are at a high, intermediate, and low risk of treatment failure, respectively. According to these groups, the proposed recommendations are as follows: salvage surgery with pathological examination, imaging in shorter intervals, or routine follow-up, respectively [112]. Campo et al., based on the results of their meta-analysis, have also suggested that the use of the repeated LB with ctHPV16 detection may represent a biomarker to complement conventional imaging-based examinations, providing a potentially useful confirmatory test in the case of positive PET-CT [70]. Some data have indicated that ctHPV16 detectable several weeks after treatment may also reflect the presence of the disease outside the treated region. Rutkowski et al. have found that, in two patients with pMR, subsequent 18F-FDG PET-CT revealed metastatic disease [78].

In summary (Table 2C), if pMR is present in at least two consecutive LBs along with a cRR, PET-CT should be considered, due to the risk of metastasis. If a cRR and cMR are observed, due to the low risk of failure, surveillance should be considered. For pRR and cMR, the repeated radiological and ctHPV16 assessment should be performed. For patients with both pRRs and pMRs, salvage surgery is indicated. 

### 5.4. ctHPV16 during Surveillance—Detection of Recurrence or Metastatic Disease 

Local recurrence occurs in approximately 20–30% of patients with HNSCC, remaining the most common cause of treatment failure. Regional recurrence in the neck occurs in 10–15% of patients, which is the next most-common cause of death in such patients. Distant metastases (DMs) are relatively uncommon, appearing in around 10% of patients [103]. Convincing data have indicated that HPV-related OPC is related to much better locoregional control and survival, compared to its HPV-unrelated counterparts [79,117]; however, a risk of locoregional treatment failure for up to 25% of patients remains [93,104,109,118]. While most recurrences of HPV-unrelated OPC occur within the first two years post-treatment, HPV-related OPC can recur up to five years after treatment, and occasional case reports have described even longer latency periods [109,119]. Of special concern is that the DM rate does not differ between HPV-related and HPV-unrelated OPC (11% vs. 15% at three years; *p* = 0.25) [109]. Interestingly, despite the comparable DM rate, the clinical scenarios and prognoses significantly differ between HPV-related and HPV-unrelated OPC patients. For HPV-related cases, the lung is the most common site of the DM, followed by the skin, brain, or intra-abdominal lymph nodes, which are equally represented [109]. Sites of metastasis that are less typical for HNSCC, such as the kidney, skin, skeletal muscle, axillary lymph nodes, and intra-abdominal lymph nodes, have also been described in HPV-related patients [120]. In HPV-unrelated OPC patients, DM occurred predominantly in the lungs. The natural course of disseminated disease presents an indolent nature for HPV-related patients. Dissemination to more than two organs was found in 33% of metastatic patients in the HPV-related OPC group, while no such scenario was observed for HPV-unrelated OPC patients [109]. As such, the survival rate after DM is significantly longer for HPV-related OPC patients [109,121]. One-fifth of all metastases may occur beyond two years, with the latest being observed 5.3 years after the completion of treatment. Therefore, active treatment interventions for DM, including surgery, radiation, and/or chemotherapy, should be considered whenever appropriate for HPV-related OPC cases [109].

Considering these observations, the detection of disease recurrence can be accelerated by ctHPV16 assessment, increasing the chances of a successful rescue. Cao et al. have reported a patient who presented Mrec only four months prior to the detection of lung metastasis [41]. In the study of Chera et al., the disease recurrence was predominantly asymptomatic and clinical examinations did not identify recurrence in any of these patients. However, 10 out of 11 patients had Mrec prior to the diagnosis of recurrence with a routine clinical follow-up. The median lead time from the first Mrec to biopsy-proven recurrence was 3.9 months (range: 0.37–12.9 months), while the radiographic detection of recurrence occurred approximately two weeks prior to the biopsy [122]. Of special issue is that, in four patients from the group with Mrec, the recurrent disease was not found because surveillance imaging CT involved only the primary tumor site (head and neck region). To avoid such a situation, the authors suggest the use of whole-body PET-CT scans in patients with two consecutive Mrec [122]. Notably, patients who develop recurrent or metastatic disease tend to have persistently elevated ctHPV, in contrast to those who present a transient elevation of ctHPV during post-treatment surveillance, which spontaneously resolves. For this reason, the Mrec should be confirmed in two consecutive assays as a criterion for identifying patients with a high risk of the recurrent disease [122].

In summary (Table 2D), MR by ctHPV16 can complement radiological assessments stratifying the risk of recurrence or metastatic disease in patients with HPV-related OPC during surveillance.

## 6. Conclusions and Future Perspectives

Detailed knowledge of the etiology of OPC is essential for precise therapy and relevant prognoses in patients suffering from this disease. In patients with active HPV-related OPC, ctHPV16 usually is present in the blood. Consequently, a ctHPV16 assay in a LB may help to confirm the HPV etiology. Moreover, a change in ctHPV16, described as MR, may support clinical decisions at all stages of the treatment and surveillance of the patients with HPV-related OPC. The assessment of MR may facilitate the modification of the ongoing treatment if necessary, early identification of treatment failure, and detection of recurrence or metastases. At present, the clinical application of MR is not a standard approach and only has supplementary value, typically being used along with other parameters such as the tissue-derived HPV etiology in primary diagnosis, radiological and clinical assessment of the treatment response shortly after treatment completion, and during surveillance. Despite the beneficial features of an LB, including its safety and repeatability, more data are clearly needed to confirm the value of ctHPV16-based MR in the prediction and/or prognosis of patients with HPV16-related OPC.

## Figures and Tables

**Table 1:** The prevalence of HR HPV genotypes.

	Mean ± SD	Median	Min–Max	References
**HPV16**	89.9 ± 3.7	89.0	84.3–96.3	(*N* = 11) 96.3% [20]; 95% [21]; 92 % [22]; 92% [23]; 90% [24]; 89% [25]; 88% [13,26,27], 86% [28]; 84.3% [29].
**HPV35**	4.1 ± 1.7	3.5	2.2–6.9	(*N* = 6) 6.9% [29], 5.1% [27], 3.6% [30], 3.4% [15], 3.1% [31], 2.2% [19]
**HPV33**	3.7 ± 2.5	3.1	1.1–7.4	(*N* = 8) 7.4% [15], 7% [13], 4.4% [29], 3.7% [27], 2.5% [31], 2.2% [19], 1.1% [20], 1.1% [30].
**HPV18**	3.0 ± 2.1	2.3	1.4–5.9	(*N* = 4) 5.9% [30], 3.1% [31], 1.5% [20], 1.4% [27]

**Table 2:** The potential role of ctHPV16-based molecular response (MR) at subsequent stages of the management of patients with HPV-related OPC.

	Phase	ctHPV16 in LB	Radiological Response	Molecular Response	Clinical Options
**A**	Prior treatment	Primary diagnosis	-	-	-
**B**	Ongoing treatment	Prediction of good response	cRR or pRR	cMR	Consider de-escalation *
**C**	After treatment completion	Assessment of early response to treatment (12 week)	cRR	cMR	Surveillance
			pRR	cMR	Another radiological evaluation
			cRR	pMR	Another LB: ctHPV16-positive: considering PET-CT ctHPV16-negative: surveillance
			pRR	pMR	Salvage surgery
**D**	Surveillance	Detection of recurrence	cRR	cMR	Surveillance
			recurrence	cMR	Biopsy or another radiological evaluation
			recurrence	Mrec	Salvage surgery
			cRR	Mrec	Consider PET-CT

* The role of de-escalation has not been proved in randomized controlled phase 3 trials. The cRR—complete radiological response; pRR—partial radiological response; cMR—complete molecular response; pMR—partial molecular response; Mrec—molecular recurrence; LB—liquid biopsy, PET-CT—positron emission tomography–computed tomography.
